# Surgeon-Centered Analysis of Robot-Assisted Needle Driving Under Different Force Feedback Conditions

**DOI:** 10.3389/fnbot.2019.00108

**Published:** 2020-01-24

**Authors:** Lidor Bahar, Yarden Sharon, Ilana Nisky

**Affiliations:** Department of Biomedical Engineering, Zlotowski Center of Neuroscience, Ben-Gurion University of the Negev, Be'er Sheva, Israel

**Keywords:** teleoperation, force feedback, needle driving, robot assisted minimally invasive surgery, learning

## Abstract

Robotic assisted minimally invasive surgery (RAMIS) systems present many advantages to the surgeon and patient over open and standard laparoscopic surgery. However, haptic feedback, which is crucial for the success of many surgical procedures, is still an open challenge in RAMIS. Understanding the way that haptic feedback affects performance and learning can be useful in the development of haptic feedback algorithms and teleoperation control systems. In this study, we examined the performance and learning of inexperienced participants under different haptic feedback conditions in a task of surgical needle driving via a soft homogeneous deformable object—an artificial tissue. We designed an experimental setup to characterize their movement trajectories and the forces that they applied on the artificial tissue. Participants first performed the task in an open condition, with a standard surgical needle holder, followed by teleoperation in one of three feedback conditions: (1) no haptic feedback, (2) haptic feedback based on position exchange, and (3) haptic feedback based on direct recording from a force sensor, and then again with the open needle holder. To quantify the effect of different force feedback conditions on the quality of needle driving, we developed novel metrics that assess the kinematics of needle driving and the tissue interaction forces, and we combined our novel metrics with classical metrics. We analyzed the final teleoperated performance in each condition, the improvement during teleoperation, and the aftereffect of teleoperation on the performance when using the open needle driver. We found that there is no significant difference in the final performance and in the aftereffect between the 3 conditions. Only the two conditions with force feedback presented statistically significant improvement during teleoperation in several of the metrics, but when we compared directly between the improvements in the three different feedback conditions none of the effects reached statistical significance. We discuss possible explanations for the relative similarity in performance. We conclude that we developed several new metrics for the quality of surgical needle driving, but even with these detailed metrics, the advantage of state of the art force feedback methods to tasks that require interaction with homogeneous soft tissue is questionable.

## 1. Introduction

Robot Assisted Minimally Invasive Surgery (RAMIS) refers to minimally invasive surgical procedure aided by robots. In RAMIS, the surgeons use a robotic manipulator to operate robotic instruments inside the patient's body. The surgical instrument (or end effector, e.g. gripper, scissors) follows the movement of the surgeon's hands, usually with different motion scaling, filtering, and other possible manipulations or restrictions. Compared to minimally invasive surgery, RAMIS has several advantages, including 7 degrees-of-freedom (DOF), 3D high definition visual system, higher precision and accuracy, and more intuitive operation (e.g., preventing the Fulcrum effect). As a result, RAMIS has the potential to produce a better surgical outcome (Garcia-Ruiz, 1998; Hagen et al., 2008; Maeso et al., 2010; Szold et al., 2015). In 2017, the number of surgical procedures using the widespread da-Vinci RAMIS system was ~877, 000 (Inc., 2017), and this number has been consistently growing over the years (Freschi et al., 2013; Enayati et al., 2016; Peters et al., 2018).

In RAMIS, surgeons that were used to operate with their hands in full contact with the patient interact with robotic interfaces that mediate between them and the patient. In this process, surgeons have lost the ability to use the crucial sense of touch, or haptic sense. The haptic sense is used in many surgical procedures for detecting lesions, navigate inside the body, and most importantly, to control the amount of force needed in each of the 7 DOF available in current teleoperation systems (De et al., 2007; Trejos et al., 2009). The haptic sense has an important role in motor control and in many surgical procedures. Currently, most commercial RAMIS systems do not offer any haptic feedback to the surgeon, although the systems are physically able to provide kinesthetic haptic feedback using embedded motors. New RAMIS systems report to have the ability to provide haptic feedback (Senhance system, TransAstrix; REVO-I Robotic Surgical System, Meere Company) (Culbertson et al., 2018; Rao, 2018).

As a consequence of the lack of haptic feedback, surgeons have to estimate the forces based only on visual information (e.g., tissue deformation, color changes of tissue) and prior knowledge (e.g., the stiffness and deformability of the specific tissue). This estimation is worsened when the surgeon's field of view is obstructed. Prior literature stressed that estimating forces and torques using other modalities can cause an inaccurate estimation, and as a consequence surgeons can apply excessive forces on the tissue ending in unwanted injuries. This drawback is especially important when considering non-expert surgeons and trainees that are in the process of learning how to control and estimate the forces correctly, and the lack of feedback is making the use of the devices less intuitive (Okamura, 2009; Johnson et al., 2014; Enayati et al., 2016).

The reasons for the lack of haptic feedback are mainly due to stability issues in closed loop teleoperation with force feedback and due to challenges in force estimation. Instability can lead to unwanted oscillations and difficulty of controlling the end effector (Enayati et al., 2016). Studies on haptic interfaces and stability have managed to give haptic feedback and ensure a stable system, but at the expense of the transparency, which is a measure of the fidelity of the teleoperation system (Lawrence, 1993; Ryu et al., 2004; Nisky et al., 2013; Enayati et al., 2016). Thus, the way the forces are rendered to the surgeon is another challenge, and understanding the effect of different conditions of force feedback on the surgeon (i.e., the movement of the operator) is a necessary step for optimizing force feedback in RAMIS.

A second reason for the lack of haptic feedback is the difficulty to estimate the forces that are applied at the end effector. Several haptic feedback algorithms were suggested to solve the challenge of force estimation. For example, position exchange is an algorithm that estimates the forces based on the error between the desired position of the robot and the actual (current) position of the robot (Siciliano and Khatib, 2016). Other studies suggested more advanced haptic feedback estimation algorithms for RAMIS (Anooshahpour et al., 2014; Dalvand et al., 2014; Li and Hannaford, 2017; Rivero et al., 2017), as well as advanced algorithms for learning the dynamics of robots that can be modified to cable-driven robots (Rubio, 2012; García-Sánchez et al., 2018; He and Dong, 2018; Rubio et al., 2018; Yen et al., 2018). Each one of the force feedback algorithms has a trade-off between system stability and transparency along with other limitations.

The benefit of haptic feedback to surgery is not yet fully understood (Okamura and Verner, 2009; Gibo et al., 2014; Yang et al., 2015; Talasaz et al., 2017). In a meta-analysis on the effect of force feedback, Weber and Schneider (2014) found that haptic feedback reduces the amount of force applied, but the reduction is smaller when depth perception is available. Another meta-analysis by Weber and Eichberger (2015) showed that the effect of force feedback is task-dependent; they found that when surgical tasks were given, the effect of force feedback was bigger, resulting in lower applied forces. The results in studies that directly compared task performance between haptic feedback condition to no haptic feedback condition were inconclusive. Mahvash et al. (2008) studied the effect of visual feedback and direct force feedback (measured by a force sensor located at the remote site) on the performance of a palpation task. They report mixed answers to the question of whether force feedback yields better performance. In a follow-up experiment, Gwilliam et al. (2009) showed that the accuracy of experienced surgeons was higher when they received haptic feedback compared to visual force feedback or no feedback. Santos-Carreras et al. (2010) compared participants' performance in needle driving in virtual reality for three different force feedback conditions: visual feedback (i.e., without haptic cues), 3 DOF force feedback and 6 DOF force feedback (forces and torques). It is important to note that the visual system in the experiment was a 2D screen (i.e., not 3D). Forces were rendered to the users by a set of equations modeling the forces in the task. In the experiment, participants performed the task using a surgical needle holder that was attached to a robotic interface. They tested three metrics—completion time, exit point error and maximum penetration depth. They show that there is a significant difference between force feedback conditions and visual feedback for exit point error and maximum penetration depth, but they did not find any benefits when adding torque feedback.

In addition to metrics that evaluate task performance, it is important to evaluate the hand kinematics of the user and characterize the learning process of users. When manipulating the tool in a teleoperation system, the kinematics are different compared to freehand movements—even when looking on simple movements such as reaching to a target (Nisky et al., 2014b), or on a more complex movement such as needle driving (Nisky et al., 2015). In a needle driving task, participants presented slower learning in teleoperation compared to needle driving that was preformed with a needle holder as in open surgery. When learning to use a teleoperation system, participants presented a learning curve over subsequent trials in path length, completion time, and other kinematic and dynamic metrics (Narazaki et al., 2006; Nisky et al., 2014b). Metrics that quantify the human movement or performance are also used to differentiate between novices and experts surgeons (Hofstad et al., 2013; Nisky et al., 2015; Sharon et al., 2017) or to evaluate the effect of training on performing surgical tasks (Judkins et al., 2008). Classical metrics, such as task completion time and path length were measured to assess the performance of participants during surgical tasks, and the learning process of surgeons with various levels of expertise (Smith et al., 2001; Nisky et al., 2015). In another study, force and torque metrics were suggested to discriminate between novice and expert surgeons (Richards et al., 2000).

In addition to classical metrics of movement and force variables, it is possible to define metrics that are based on research of the motor system. The motor system is complex and the different levels of movement execution are subjects of numerous studies (Kandel et al., 2000; Flash et al., 2013). To execute even the simplest movement our motor system is engaged in a series of tasks that end in the desired movement. Due to redundancy at many levels of the system, there are many ways the motor system can perform the same task. As a result, modeling the motor system and its movements is a non-trivial task (Franklin and Wolpert, 2011; Jarc and Nisky, 2015, 2020). One prominent kinematic law of the human movement is the two-thirds power law for planar trajectories and its generalization to 3 dimensional movements, known as the one-sixth power law. The two-thirds power law describes the relation between movement's curvature and speed, and the one-sixth power law adds dependency on torsion. Several studies have shown that the law applies for simple and complex movements (Pollick et al., 2009; Flash et al., 2013). Sharon and Nisky (2018) showed that surgeon expertise and the use of teleoperation effects the parameters of the one-sixth power law. Another suggested principle in human movement is the minimum jerk. Jerk is the 3rd derivative of the position, and studies showed that human movement is optimized such that its jerk will be minimal (Flash et al., 2013). Jerk is used to measure to the smoothness of the movement: lower jerk is smother movement. Several ways were suggested to quantify the smoothness of the human movement (Hogan and Sternad, 2009; Balasubramanian et al., 2012; Estrada et al., 2016; Pandey et al., 2017).

In order to study how haptic feedback affects the motor performance in a needle driving task and specifically how it affects the learning of the task, we quantified the performance as well as the different aspects of the movements using classic and novel metrics. We developed metrics for evaluation of the motor system performance in the needle driving task. Using the metrics and trial by trial experiment with different force feedback conditions, we assessed the effect of the different haptic conditions on learning and performance.

## 2. Materials and Methods

### 2.1. Needle Driving Task

**Figure 1:**
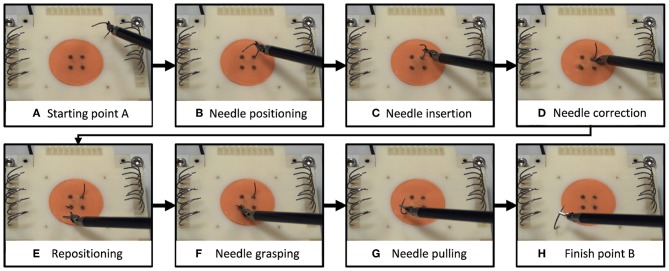
Needle driving task. We divided the needle driving task into 8 subtasks **(A–H)**. On each trial, participants **(A)** grasped a needle and positioned it above the starting point, **(B)** transported the needle to the insertion point and positioned it, and **(C)** inserted the needle into the tissue (1st insertion). **(D)** If needed, participants repositioned the tool on the needle and continued in insertion (2nd insertion). Then they **(E)** repositioned the tool on the needle for extraction, **(F)** grasped the needle for extraction, **(G)** extracted the needle, and **(H)** positioned the needle above the finish point.

We selected a surgical needle driving task for this experiment, and designed an experimental setup to characterize participants' movements and applied forces. Needle driving encompasses a complex movement (movement in 6 DOF, divided into several subtasks) combined with interaction with soft tissue and it is a common procedure in surgeries. Tissue interaction forces can add important information to the operator when interacting with a soft object. The goal of needle driving is driving a surgical needle between two points through tissue using a surgical tool. The participants were requested to drive the needle while following the arc of the needle with the tooltip, maintaining tool tip velocity direction perpendicular to the needle curve. We divided the needle driving task into six subtasks (Figure 1), (a) needle positioning, (b) needle insertion, (c) needle correction (optional), (d) repositioning, and (e) needle pulling. We refer to needle correction as needle insertion after repositioning the tool on the needle; therefore, this subtask is optional, and the participants were instructed to avoid corrections as much as possible.

### 2.2. Experiment Setup

#### 2.2.1. da-Vinci Research Kit—Hardware and Control

**Figure 2:**
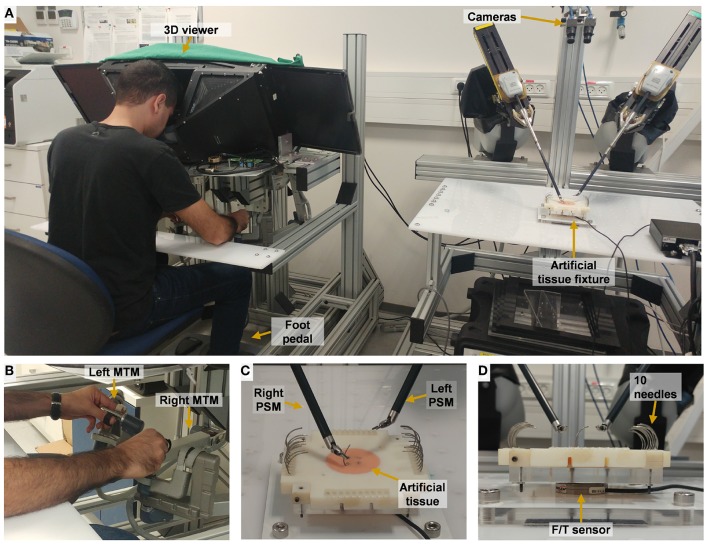
Experimental setup for teleoperation. **(A)** The participant uses the two dVRK Master Tool Manipulators (MTM) to control the patient side tools. 2 HD cameras are used to acquire the visual information that is presented to the user. **(B)** Master side: the participant manipulate left MTM and right MTM. **(C)** Patient side: needle driving is performed on the artificial tissue using the right tool of the patient-side manipulator (PSM). **(D)** A side view of the artificial tissue fixture. Forces and torques were recorded by the F/T sensor that is embedded in the fixture.

The experimental apparatus was the da-Vinci Research Kit (dVRK) (Kazanzides et al., 2014). The dVRK is based on first generation da-Vinci system hardware (Intuitive Surgical Inc.), and has open hardware controllers and open source code (Kazanzides et al., 2014; Chen et al., 2017). The dVRK teleoperation setup (Figure 2) included two Master Tool Manipulators (MTM), two Patient Side Manipulators (PSM), 4 controllers (one for each manipulator), foot pedals tray and high resolution stereo viewer. During teleoperation, the patient side manipulators (PSMs) follow the movement of the master manipulators (MTMs) after scaling and proper changes to orientation. Each MTM is capable of providing 6 DOF haptic feedback. In this experiment, we used 2 large needle drivers as patient side instruments. The 3D vision system consists of 2 HD cameras (FLIR Blackfly S cameras, BFS-U3-32S4C, 3.2 MP; Edmund Optics Lenses, 16 mm, f/1.8 Ci Series Fixed Focal Length Lens), and 2 HD flat screens (frame rate of 35 Hz, resolution of 1080 × 810). The dVRK controllers were connected to Ubuntu (UNIX) OS computer with an Intel Xeon E5-2630 v3 2.40 GHz processor. The vision system was connected to Ubuntu OS computer with Intel Core i7-7700K 4.2 GHz processor and NVIDIA Quadro P2000 5 GB graphics card. The communication between the two computers was done over the university's local area network (LAN) and ROS multiple machines configuration. The maximal latency that was measured using this communication was 0.5 ms.

**Figure 3:**
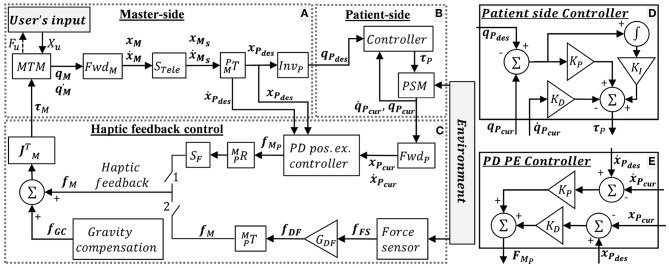
dVRK teleoperation control. **(A)** Master-side, **(B)** patient side, and **(C)** haptic feedback conditions: when switch 1 is closed, the user receives position exchange (PE) haptic feedback. When switch 2 is closed, the user receives direct feedback (DF) haptic feedback. When both switches are open, the user does not receive feedback (NF). In addition, dVRK gravity compensation (GC) is enabled. **(D)** Patient side dVRK controller (presented in **B**) **(E)** PD PE controller for the PE haptic feedback. Complete description is given in section 2.2.1.

The control mechanism of the dVRK teleoperation is depicted in Figure 3, where *x* and ẋ denotes the end-effector's Cartesian space position and velocity, respectively, and *X* denotes a vector of *x* and ẋ. *q* and $\overset{∙}{q}$ denotes the joints' position and velocity, respectively. M, P, and u denote MTM, PSM and user, respectively. “des” and “cur” denotes desired and current. R, T, S, and J denotes rotation matrix, transformation matrix, scaling and Jacobian, respectively. Forward and inverse kinematics are denoted by Fwd and Inv, respectively. *f* and τ denotes Cartesian forces and motor torques, respectively. The user moves the MTMs and receives 3DOF haptic feedback. The patient side (Figure 3B) follows the master side (Figure 3A) with proper scaling and transformation. The scaling *S*_*Tele*_ = 0.4 was chosen such that the user reaches the entire experimental workspace. The PSM control is depicted in Figure 3D. The PSM interacts with the environment (i.e., needle, tissue).

When switch 1 is closed, the user receives position exchange (PE) haptic feedback. In PE haptic feedback, the PD controller (Figure 3E) receives desired and current position and velocities, such that the error between them is used to calculate the force feedback. The *K*_*P*_ and *K*_*D*_ for the PD PE controller were tuned manually to roughly match the output forces to the forces recorded by the Force/Torque (F/T) sensor. The equation for the PD PE controller is:

(1) $$\begin{array}{r} f_{Mp}=K_{D}\left(x_{P_{des}}-x_{P_{cur}}\right)+K_{P}\left({\overset{∙}{x}}_{P_{des}}-{\overset{∙}{x}}_{P_{des}}\right) \end{array}$$

where ***x*_*P*_*des*__** and ***x*_*P*_*cur*__** are the desired (based on the position of the MTM) and current positions of the PSM, respectively, ${\overset{∙}{x}}_{P_{des}}$ and ${\overset{∙}{x}}_{P_{des}}$ are the desired and current velocities of the PSM, and *K*_*P*_ and *K*_*D*_ are the positional and derivative gains of the PD controller. The values are $K_{P}=1000\frac{kg}{s^{2}}$ and $K_{D}=30\frac{kg}{s}$.

When switch 2 is closed, the user receives direct feedback (DF) haptic feedback. The equation for the DF haptic feedback is:

(2) $$\begin{array}{r} f_{DF}=G_{DF}\cdot f_{FS} \end{array},$$

where, ***f*_*FS*_** is the force measured by the force sensor, and *G*_*DF*_ is the gain of the force. The DF feedback is sampled by the force sensor in 500 Hz and is transmitted to the operator directly with a gain of *G*_*DF*_ = 0.7. We chose the 0.7 force scaling empirically as the largest gain in which participants were able to easily stabilize the system. Maximum latency in DF haptic feedback was ~20 ms. We measured the latency experimentally by using left PSM to invoke the F/T sensor direct feedback to the right PSM. This way we could find the difference between the positions of both PSMs and measure the maximum latency. In both force feedback conditions, the forces were rendered to the user at 500 Hz. When both switches are open, the user did not receive feedback (NF). Both switches were never closed together. In addition, dVRK gravity compensation (GC) was enabled (Kazanzides et al., 2014; Chen et al., 2017).

#### 2.2.2. Open and Teleoperation Setup

**Figure 4:**
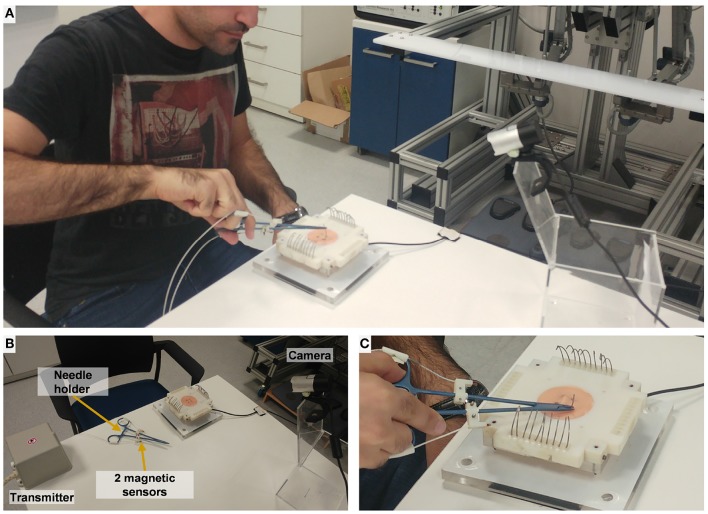
Experimental setup for open needle driving. **(A)** The participant performs open needle driving using a titanium needle holder. **(B)** Magnetic transmitter records 6 DOF pose of the 2 magnetic sensors. **(C)** Artificial tissue, fixture, and F/T sensor setup (identical to the teleoperated conditions).

The experimental setup included two parts: (1) open needle driving, as in open surgery, and (2) teleoperated needle driving with the dVRK. The open needle driving setup (Figure 4) consisted of magnetic tracking sensors (TrakStar, Ascension Technologies, NDI), a titanium needle holder (Fine Needle holder, 16 cm, Serrated, Titanium, World Precision Instruments), and a single HD camera (LifeCam Studio 1080p FHD WebCam, Microsoft). We instrumented the surgical needle holder with two magnetic 6DOF sensors. The sensors were mounted on the needle holder using custom made 3D printed fixtures that attached the sensors to the two rods of the needle holder. We calculated the tooltip position and orientation using the two magnetic sensors' position and orientation. The teleoperated needle driving setup (Figure 2) included the dVRK, 3D HD cameras and two large needle driver tools (Intuitive Surgical). The foot pedal had to be pressed to enable teleoperation.

For both setups (open and teleoperated), we designed a 3D-printed custom fixture for the artificial tissue. The artificial tissue was made from a silicone molded homogenous piece (EcoFlex 00-30 mixed with 10% Silicone Thinner, Smooth-On inc.). The tissue was molded around 2 wooden sticks that were used to fix the tissue to the 3D printed fixture. The fixture and tissue were mounted on an acrylic plate that was fixed to a force/torque sensor (Nano 43 F/T sensor, ATI Industrial Automation) that measured tissue interaction forces and torques. The sensor was mounted beneath the tissue (see Figure 2D), and its vertical axis (Z) was normal to the face of the tissue. The 3D-printed fixture could be rotated in 4 different right angles, to enable rotation of the tissue between the experimental blocks to allow for working on a fresh portion of the artificial tissue in each block. We used surgical needles for general surgery (GS-21, Covidien), 10 needles were placed on the fixture in each block for participants to use.

### 2.3. Experimental Procedures

Thirty participants (*N* = 30) took part in the experiment after signing an informed consent form. The protocol and the form were approved by the Human Subject Research Committee of Ben Gurion University of the Negev, Beer-Sheva, Israel. All participants were right-handed, and they used the right needle driver to perform the needle driving, and the left needle driver (or their left hand in the open setup) to adjust the needle as needed.

**Figure 5:**
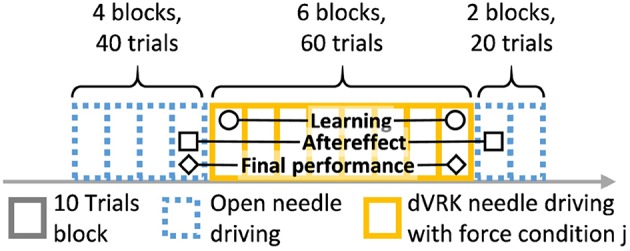
Experimental protocol. Participants performed 120 trials of needle driving that were divided into 12 blocks. In our statistical analysis, we focused on 3 contrasts: learning, aftereffect and final performance. Learning: the effect of practice in teleoperation—the difference in performance between early and late teleoperation. Aftereffect: the effect of teleoperation on open needle driving—difference in performance between just before and just after teleoperation. Final performance: the performance at the end of teleoperation compared to the participant's baseline—the difference between the late teleoperation performance and the performance just before teleoperation.

Participants were asked to perform 120 trials of needle driving with the open and the teleoperated setups. The experimental protocol is depicted in Figure 5. The protocol was divided into 12 blocks, and each block included 10 trials. After each block, the artificial tissue was rotated such that a fresh entrance and exit point were presented to the participant to prevent wearing of the silicone tissue. The needle driving desired path was not changed, the tissue fixture was rotated by 90 degrees in the horizontal plane. All the participants first performed 40 trials of open needle driving. We used the performance of each participant at the end of this baseline open part to compare with their performance at the subsequent parts of the experiment. In the second part of the experiment, all the participants performed 60 trials of needle driving task using the dVRK. Each group received one of three feedback conditions: (1) no haptic feedback (NF, *N* = 10), (2) direct sensing from a force sensor that was mounted under the tissue (DF, *N* = 10), and (3) position exchange based force feedback (PE, *N* = 10). After completing the teleoperation part, all the participants performed additional 20 trials using the open needle driving setup. This transition back to performing open needle driving after teleoperation represents a scenario that could happen in real life surgeries in case that the surgeon decides that the procedure cannot be safely completed with robotic assistance, and is used to assess the aftereffect of teleoperation with different feedback conditions on the performance in open needle driving.

Prior to the first part of open needle driving, and prior to the second part of teleoperation needle driving, participants viewed a video with instructions on how to perform the task correctly using the needle holder and using teleoperation, respectively (as in Nisky et al., 2015). Participants were asked to perform the needle insertion in one throw, and if needed, they could readjust their gripping of the needle and continue the insertion along the same curve of the needle. In addition, they were asked to work as quickly and as accurately as possible. Then, participants were asked to do a short practice. Before the open needle driving, participants practiced the use of the needle holder together with the surgical needle (without interaction with the tissue). Before the teleoperation part, participants were asked to adjust the system to their comfort (adjust chair and vision system's height and 3D vision of the environment was validated). Afterward, participants practiced several teleoperation exercises, to familiarize themselves with the dynamics of the robot. They were asked to: (1) move in a circle around the tissue and upwards, (2) reach in all directions—forwards, backward and to the sides, (3) touch the tissue and fixture using the teleoperation tool, (4) pick up the needle and release, and (5) pantomime the needle driving movements without touching the tissue.

After completing this practice, they began the test trials. After the first three trials of the open needle driving and first three trials in teleoperation needle driving part, participants received feedback on correct and incorrect performance; Emphasis was given on simultaneous wrist rotation during needle insertion and extraction, correct needle gripping, correct use of needle holder, and insert and exit of the needle at desired points. After each block, the tissue fixture was rotated, and participants received a short break.

### 2.4. Data Analysis

#### 2.4.1. Sampling and Preprocessing

In the open needle driving setup, we recorded the data at 120 Hz, and in the teleoperation setup we recorded at 500 Hz. We down-sampled and interpolated all the signals to 100 Hz. Both position and force data were interpolated using the shape-preserving piecewise cubic interpolation. Orientation data was interpolated using the spherical linear quaternion interpolation method (Slerp). We filtered tooltip position data with a 2nd order Butterworth low pass filter with cut-off frequency of *F*_*c*_ = 10 Hz using Matlab *filtfilt()* function, resulting in zero phase filtering with a cut-off frequency of *F*_*c*_ = 8 Hz. Velocity, acceleration, and jerk were calculated using numerical differentiation. After each differentiation, we filtered the signal using the same filter that was used for the tooltip signal.

#### 2.4.2. Segmentation

We defined trial start as the first interaction of the needle with the tissue, and the end of the trial as the final interaction with the tissue. We automatically segmented trial start and end based on force and tooltip position samples—the first and last samples for which the force was higher than the noise threshold (*th* = 0.06[*N*]), and the tooltip position was inside the artificial tissue contour (i.e., there was interaction with the tissue, not with the fixture). We manually validated this trial segmentation based on force profiles and recorded trial video, and corrected erroneous segmentation due to accidental tissue interaction. Corrected segmentation samples were selected from a pool of samples that was marked by the automatic segmentation algorithm as possible segmentation samples.

We also automatically segmented each trial into trial subtasks (Figure 1). The automatic segmentation algorithm was designed based on task definition and based on the unique characteristics of the setup. When the gripper was closed and tissue interaction forces were above the noise threshold, the data point was classified as belonging to one of the needle driving segments (insertion, correction, or extraction). Insertion was defined as the first sequence of data points that belonged to the needle driving segments. Correction segments were classified if tooltip position was closer to needle entrance point rather than exit point. Extraction segments were defined as last sequence of data points belonging to the needle driving segment, or classified when tooltip position was closer to needle exit point rather than entrance point.

#### 2.4.3. Metrics of Performance

We quantified participants performance using four classes of metrics: (I) task performance, (II) forces, (III) kinematics, and (IV) motor control grounded metrics. In the following section, we will review all the metrics that we used, including classical metrics and novel metrics that we developed in this study.

We used two metrics to quantify task performance. *Task completion time* was calculated as the cumulative time in which participants preformed the task:

(3) $$\begin{array}{r} \text{Completion Time}=t_{end}-t_{start} \end{array},$$

where, *t*_*start*_ and *t*_*end*_ are the start and end time of the task, respectively. *Exit point error* metric was measured as the Euclidean distance between desired exit point and actual exit point:

(4) $$\begin{array}{r} \text{Exit Point Error}=∥x_{desired}-x_{actual}∥, \end{array}$$

where ***x*_*desired*_** and ***x*_*actual*_** are vectors of the *x, y* data point in the plane of the tissue surface. The data of the actual and desired exit point was extracted using the images recorded by the cameras. Since the cameras and tissue in the teleoperation setup are fixed and identical between all trials, we could use the known dimension of the tissue to calculate the distance between them. Because the camera in the open needle driving setup was not fixed, we could not use the images to extract the exit point error, thus the exit point error metric was measured in the teleoperation needle driving setup only.

We used five metrics to quantify the forces that the participants applied on the tissue. We calculated the *total normalized force* as:

(5) $$\begin{array}{r} \text{Total Normalized Force}=\frac{1}{d_{ie}}\overset{N-1}{\underset{n=1}{\sum }}|f\left(n\right)|\cdot \Delta t\left(n\right), \end{array}$$

where |***f***| denotes the total force, Δ*t*(*n*) = *t*(*n*) − *t*(*n* − 1) is the time difference between consecutive samples, N is the number of samples, and *d*_*ie*_ is the Euclidean distance between actual entrance and exit points. Since we used the Euclidean distance that was extracted only from the camera in the teleoperation setup, this metric is calculated only in the teleoperation setup. The metric quantifies the total forces that participants applied on the tissue as an indication of damage to the tissue. The rationale is that some amount of force is needed for the needle driving itself, but if there are substantial forces that were applied in incorrect directions the total cumulative forces will be higher. However, if the participants traveled a longer path inside the tissue due to inaccurate performance of the needle driving, larger forces will accumulate as well. Therefore, we normalized the cumulative forces by the distance traveled through the tissue.

Additional metric that quantified forces is the maximum force applied on the tissue:

(6) $$\begin{array}{r} \text{Max Force}=\max\left(\left|f\right|\right), \end{array}$$

where |***f***| denotes the tissue interaction forces. The maximum torque around Z axis was calculated as:

(7) $$\begin{array}{r} \text{Max TorqueZ axis}=\max\left(\tau_{z}\right), \end{array}$$

where τ_*z*_ denotes the torque around the vertical axis. This metric ideally should be zero, since the optimal needle driving movement is planar, and the needle should not rotate around the normal to the tissue direction.

The *force consistency* of consecutive movements was calculated as the sum of squared Euclidean cumulative distance between all force profiles to their mean profile:

(8) $$\begin{array}{r} \text{Force Consistency}=\frac{1}{N_{t}}\overset{N_{t}}{\underset{i=1}{\sum }}\overset{N-1}{\underset{n=0}{\sum }}{\left(|f_{i}\left(n\right)-f_{mean}\left(n\right){|}_{2}\right)}^{2}, \end{array}$$

where ***f*** is the 3DOF tissue interaction forces, *i* denotes force profile index, and N is the number of samples within a single trajectory, and *N*_*t*_ = 5 is the number of trajectories. Prior to the calculation of this metric we aligned the force trajectories using Dynamic Time Warping (DTW) to find the mean trajectory and to allow for calculating the distance between two trajectories.

We used five metrics to quantify movement kinematics to assess movement efficiency and consistency. We calculated the *path length* of the movement as:

(9) $$\begin{array}{r} \text{Path Length}=\overset{N-1}{\underset{i=1}{\sum }}|x_{i+1}-x_{i}{|}_{2},\; \end{array}$$

where *N* is the number of samples. This is a classical metric (Smith et al., 2001; Narazaki et al., 2006; Hofstad et al., 2013; Nisky et al., 2014b; Nisky et al., 2015), and lower values imply higher efficiency of the movement.

**Figure 6:**
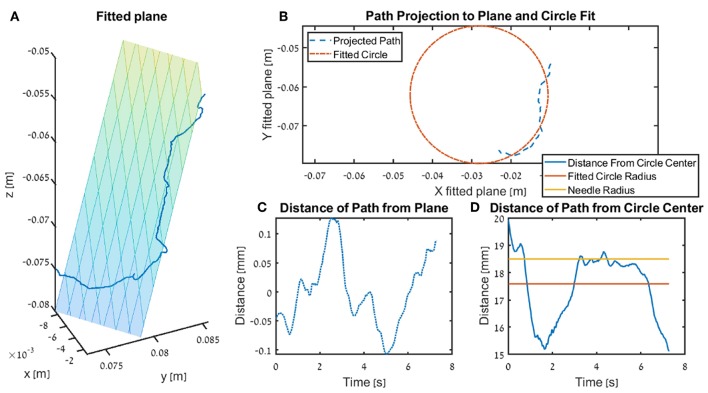
Illustration of the fitted plane and circle use in the kinematics metrics *circle deviation* and *plane deviation* for a single trial. In the figure we present the insertion subtask for one participants that received DF feedback, and the trial is recorded from the teleoperation setup. **(A)** The fitted plane and the path. **(B)** An example of a projected path (seen in 3 dimension in **A**) and the circle fitted to that path. **(C)** The distance of the path to the plane (i.e., shortest distance of each point). **(D)** The radius of the surgical needle (yellow) used in the experiment, together with the radius of the fitted circle (red) and the distance of the tooltip path from the center of the fitted plane.

We calculated three metrics that quantify how close the participants were to performing the needle driving according to the instructions. For each trajectory, we fitted a circle that ideally should be with an identical diameter to that of the needle, and any deviation of the tip of the needle driver from that circle results in a movement that does not push the needle along its arc. We calculated the *Circle deviation* as the integrated distance of the projected path from the best fitted circle, normalized by the length of the projection of the path on the curve of the circle (see ilustration in Figure 6).

(10) $$\begin{array}{r} \text{Circle Deviation}=\frac{1}{\Theta_{arc}}\overset{N-1}{\underset{n=1}{\sum }}s^{2}\left(n\right)\cdot \Delta \theta \left(n\right), \end{array}$$

where, *s* is the Euclidean distance between the fitted circle and the path projected on the fitted plane, Δθ(*n*) = θ(*n*) − θ(*n* − 1) is the angle difference between consecutive samples of the tool tip on the arc of the fitted circle, N is the number of samples, and Θ_*arc*_ = *r*_*needle*_Δθ_*arc*_ is the total arc path in meters, where Δθ_*arc*_ is the difference between the angle of the tool tip at the beginning of the movement to the angle at the end of the movement on the arc of the fitted circle.

The *Plane deviation* quantifies the cumulative deviation from the movement plane as the integrated distance of the trajectory from the plane that is fitted to the movement, normalized by the length of the path (see ilustration in Figure 6). We fitted the plane to the hand path using Matlab *fitlm()* function, we used the vertical axis (*z* axis) as the dependent variable, and the horizontal axes (*x, y*) as the independent variables.

(11) $$\begin{array}{r} \text{Plane Deviation}=\frac{1}{PL}\overset{N-1}{\underset{n=1}{\sum }}r^{2}\left(n\right)\cdot \Delta x\left(n\right), \end{array}$$

where, *r* is the Euclidean distance between the fitted plane and the trajectory, Δ*x*(*n*) = |***x*(*n*)** − ***x*(*n* − 1)**|_2_ is the path length between consecutive samples of the tool tip, N is the number of samples, and *PL* is the total path length in meters.

The *normalized angular path* (Equation 12) quantifies wrist rotation during needle driving (Sharon et al., 2017).

(12) $$\begin{array}{r} \text{Normalized Angular Path}=\frac{1}{PL}\overset{N-1}{\underset{i=1}{\sum }}\Delta \theta_{i,i+1}, \end{array}$$

where, PL is the path length, Δθ_*i, i*+1_ is the angular rotation between two consecutive samples, and N is the number of samples. This metric was suggested in Sharon et al. (2017), where higher normalized angular path was associated with expertise and with learning of the needle driving with repeated performance of the task.

The *trajectory consistency* (Equation 13) of consecutive movements was calculated as the sum of squared Euclidean cumulative distances between all trajectory profiles to their mean profile. We aligned trajectory profiles using Dynamic Time Warping (DTW) (computed by Matlab *dtw()* function with the *squared* metric) to find the mean trajectory and to calculate the distance between each pair of trajectories.

(13) $$\begin{array}{r} \text{Trajectory Consistency}=\frac{1}{N_{t}}\overset{N_{t}}{\underset{i=1}{\sum }}\overset{N-1}{\underset{n=0}{\sum }}{\left(|x_{i}\left(n\right)-x_{mean}\left(n\right){|}_{2}\right)}^{2},\; \end{array}$$

where, ***x*** is the 3 DOF position, *i* denotes trajectory index, and N is the number of samples within a single trajectory, and *N*_*t*_ = 5 is the number of trajectories.

We used motor control grounded metrics to quantify to what extent the movements of the participants satisfy known laws in human motor control that were proposed for simpler movements. We focused on the speed-curvature-torsion power law (Pollick et al., 2009; Sharon and Nisky, 2018) and the minimum jerk (Hogan and Sternad, 2009). The speed-curvature-torsion power law is defined as:

(14) $$\begin{array}{r} v=\alpha \kappa^{\beta }|\tau {|}^{\gamma }, \end{array}$$

where *v* is the speed, α is the velocity gain factor, κ is the curvature and τ is the torsion. β and γ are the power constants, and in scribbling movements they were proposed to be equal to −1/3 and −1/6, respectively (Pollick et al., 2009) (but may be different in needle-driving, Sharon and Nisky, 2018). We used linear regression on the log-transformed data to fit the power law to the participants trajectories, and calculated the optimal α, β, and γ estimates.

In addition, we calculated the root mean squared jerk (Equation 15) of participants' movements.

(15) $$\begin{array}{r} \text{Jerk}=\sqrt{\frac{1}{t_{2}-t_{1}}\overset{N-1}{\underset{n=1}{\sum }}{\dddot{x}\left(n\right)}^{2}\cdot \Delta t\left(n\right)},\; \end{array}$$

where, $\dddot{x}$ is the jerk of the movement, Δ*t*(*n*) = *t*(*n*) − *t*(*n* − 1) is the time difference between consecutive samples, and N is the number of samples. Jerk is a measure for movement smoothness, the lower the jerk, the smoother the movement. There are several suggested methods to calculate the jerk of a movement (Hogan and Sternad, 2009). Following an analysis of the different methods on our data, we chose the method that normalizes the jerk by movement duration, and has units of jerk. As a result, this jerk metric is less correlated with completion time. Because this metric spans over several orders of magnitudes, we chose to analyze the logarithm of the metric.

#### 2.4.4. Statistical Analysis

In our statistical analysis we compared three contrasts (Figure 5): learning in teleoperation, aftereffect, and final performance. In the learning contrast, we analyze the effect of practice in teleoperation: the difference in performance between early and late teleoperation. We define early and late as the median of the first 5 trials and median of last 5 trials in teleoperation, respectively. In the aftereffect contrast, we analyze the effect of teleoperation on open needle driving: the difference in performance between just before (last 5 trials of 1st open needle driving) and just after teleoperation (first 5 trials of 2nd open needle driving). In the final performance contrast, we analyze the performance at the end of teleoperation compared to participants baseline: the difference between the late teleoperation (last 5 trials in teleoperation needle driving) performance and the performance just before teleoperation (last 5 trials in 1st open needle driving). We chose to use the medians of blocks of 5 trials in our statistical analysis to reduce the effect of trial-by-trial variability in the performance of the participants.

Most of our metrics did not distribute normally. Therefore, we chose the median of each block as the statistic to quantify the metrics values. We used non-parametric statistical methods. For each one of the contrasts above, we used the two-sided Wilcoxon sign rank test to determine whether the difference between the two stages in each contrast was statistically different from zero. In addition, for each contrast separately, we used the Kruskal-Wallis test to determine whether we can reject the alternative hypothesis that the metrics describing the three force feedback conditions belong to the same distribution. Since none of the KW tests showed statistical significant differences no *post-hoc* tests were needed.

## 3. Results

To assess the performance of our participants in the needle driving task, we first visually examined the recorded trajectories of the tissue interaction forces and the kinematics of the patient side instrument tip. We compared the trajectories in the different dVRK teleoperation conditions and the trajectories in the open needle driving. Following the visual examination, we quantified the performance of the participants using novel and classical metrics. We grouped the metrics into the following categories: task performance, tissue interaction forces, kinematics, and motor control grounded metrics. Because most of the metrics were not normally distributed, we used non-parametric statistical tests to assess the effect of feedback conditions on each one of the metrics. Specifically, we focused on the effect of the different feedback conditions (no feedback NF, position exchange PE, and direct force feedback DF) on (i) the *learning* during teleoperation, (ii) the *final performance* in teleoperation, and (iii) the *aftereffect* of teleoperation on needle driving using an open surgical needle holder.

**Figure 7:**
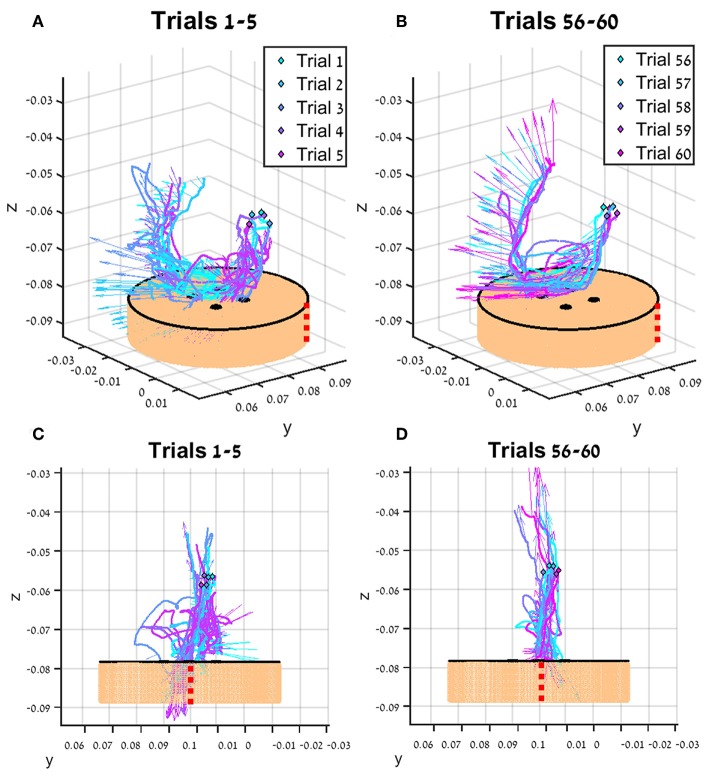
An example of trajectories (lines) and tissue interaction forces (arrows) from the first 5 teleoperated trials **(A)** and last 5 trials **(B)** of participant from direct force (DF) feedback condition. The start of each trajectory is denoted by a diamond marker. Tissue interaction forces are represented by arrows, and each arrow represents the direction of the force at the specific sample together with the size of the force (length of the arrow). Different viewpoint of the same trajectories are presented in **(C,D)**. The skin-toned flat cylinder represents the mock tissue specimen at scale. Red dotted line is presented to orient the viewer in space.

**Figure 8:**
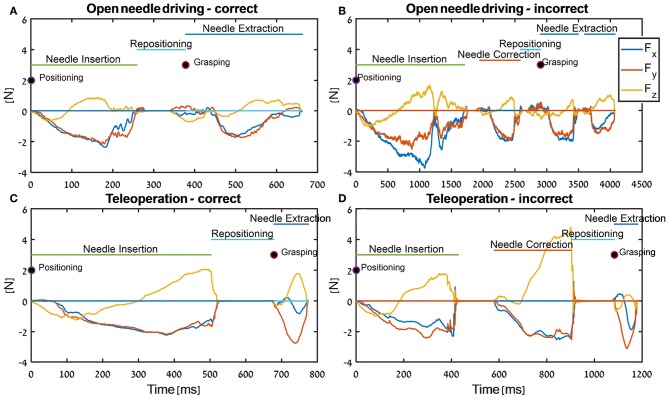
Examples of force trajectories in each axis during needle-driving trials in open needle driving **(A,B)** and in teleoperation **(C,D)**. We present force trajectory for a trial that was preformed correctly (open needle driving - **A**, teleoperation - **C**) and for a trial that was preformed incorrectly (open needle driving - **B**, teleoperation - **D**). In addition, the segments that were identified by the automatic segmentation algorithm are annotated.

In Figure 7, the tissue interaction forces and translation trajectories of a single participant in the first 5 trials (Figures 7A,C) and the last 5 trials (Figures 7B,D) are depicted. Visual examination of the force and translation trajectories suggests that the last 5 trials are more consistent (i.e., similar to each other) compared to the first 5 trials. Moreover, the trajectories seem to be less jerky and more planar (Figures 7C,D). In Figure 8, Examples of tissue interaction forces as a function of time during a single trial in open needle driving (Figures 8A,B) and teleoperation (Figures 8C,D) are presented. When performing the task correctly (Figures 8A,C), we identified a pattern in the tissue interaction forces. In the insertion part, the ideal driving trajectory is oriented along the *x-y* diagonal, and indeed the needle horizontal (*x-y* plane) forces are correlated. The vertical (*z*) component of the force vector is oriented in the negative vertical direction when the needle first penetrates the tissue, but the direction is reversed when the needle starts pointing toward the extraction point. During repositioning, there are occasional small forces due to transient interactions with the tissue. The extraction is a single fast movement of pulling the needle both in the horizontal plane and in the vertical axis, and as the movement progress, the vertical force component is more dominant compared to forces in horizontal plane. This pattern was consistent across participants in successful needle driving trials. For reference, examples of two trajectories of less successful trials are depicted in Figure 8B for open needle driving and Figure 8D for teleoperation. For both trajectories, participants did not succeed to insert the needle in one throw and needed a second throw to correct. The maximum tissue interaction force of the teleoperation example is higher compared to the correct example, and for the open needle driving the force trajectories have a different pattern and are less smooth.

In our analysis, we divided the needle driving task to several subtasks, and present here the results only for the insertion subtask. We focused on the *insertion* subtask because it is most important for the success of the overall needle driving, and its relative duration is highest (median relative duration and standard deviation of 50.9%±10 for insertion; 16.6%±14 for extraction; 16%±14 for repositioning) compared to the other subtasks.

To compare participants, performance between the three feedback conditions, we quantified three contrasts: (1) the *learning during teleoperation* (tele late—tele early) is presented in **Figure 10**, (2) the *aftereffect* of each teleoperation condition on open needle driving (open 2 early—open 1 late) is presented in **Figure 12**, and (3) the *final performance* of each participant in teleoperation compared to the participant's baseline performance (tele late—open 1 late) is presented in **Figure 11**. For each metric, we present the median of the difference of each contrast and a non-parametric bootstrap 95% confidence interval for each of the metrics in each of the teleoperation conditions, along with gray markers denoting individual participants. Statistical results of all metrics for the three contrasts are presented in two tables: Wilcoxon sign rank test results are summarized in Table 1 and Kruskal-Wallis test results are summarized in Table 2. In these tables, statistically significant values are marked with bold letters.

**Table 1:** Statistical analysis—Wilcoxon sign rank.

**Metric name**		**Learning**			**Aftereffect**			**Final performance**		
		**NF**	**PE**	**DF**	**NF**	**PE**	**DF**	**NF**	**PE**	**DF**
Exit point error	p	0.37	0.06	0.23	–	–	–	–	–	–
	Δ	0.50	0.95	1	–	–	–	–	–	–
Completion time	p	**0.027**	**0.0019**	**0.0058**	0.064	0.92	1	**0.0058**	0.43	**0.037**
	Δ	**1.99**	**3.8**	**5.47**	−0.50	−0.079	−0.13	**−1.62**	−1.02	**−1.64**
Total Force normalized	p	**0.013**	**0.0039**	**0.0019**	–	–	–	–	–	–
	Δ	**0.46**	**0.66**	**0.98**	–	–	–	–	–	–
Max force	p	0.77	0.13	0.32	**0.019**	0.84	0.43	**0.0039**	**0.037**	0.84
	Δ	−0.34	0.70	0.79	**0.35**	−0.053	0.23	**−1.37**	**−0.72**	0.11
Max torque - Z axis	p	0.69	**0.0039**	0.10	0.32	0.10	0.064	0.37	0.16	0.55
	Δ	−3	**7.9**	5.2	3.03	11	6.87	6.54	11.3	9.36
DTW - force	p	0.19	**0.027**	**0.037**	0.84	0.43	0.10	**0.002**	**0.013**	0.19
	Δ	451	**1330**	**2140**	16.6	−83.2	175	**−968**	**−364**	−192
Path length	p	0.084	0.10	**0.019**	0.23	**0.037**	1	0.37	**0.0097**	0.69
	Δ	0.010	0.020	**0.031**	0.0082	**0.010**	0.0010	0.011	**0.023**	0.0020
Circle deviation	p	0.27	0.16	0.43	0.23	0.064	0.84	0.37	**0.037**	0.84
	Δ	4.78·10^−6^	3.20·10^−6^	1.20·10^−6^	5.84·10^−6^	4.94·10^−6^	8.82·10^−7^	5.79·10^−6^	**7.10·10^−6^**	4.34·10^−7^
Plane deviation	p	**0.027**	**0.013**	0.55	0.84	0.19	0.55	0.43	**0.037**	0.69
	Δ	**1.85·10^−6^**	**2.16·10^−6^**	1.75·10^−6^	−4.81·10^−7^	1.82·10^−6^	−8.20·10^−7^	8.62·10^−7^	**2.64·10^−6^**	−1.80·10^−7^
Angular path	p	0.10	0.55	0.27	0.69	0.92	0.13	0.77	0.16	0.16
	Δ	−5.96	3.53	−9.44	−10.7	1.32	15.5	8.15	−7.89	16.6
DTW - position	p	0.10	**0.019**	**0.027**	0.77	0.43	0.92	0.32	0.62	0.10
	Δ	0.010	**0.020**	**0.026**	0.0021	−4.28·10^−5^	0.0014	−0.0042	−0.0010	−0.0041
1/6 power law α	p	0.32	0.084	0.13	0.77	0.32	0.23	**0.013**	**0.0097**	0.084
	Δ	0.035	0.038	0.059	0.016	0.038	0.049	**0.083**	**0.15**	0.061
1/6 power law β	p	0.32	**0.013**	0.32	0.92	0.77	0.37	0.77	0.13	0.13
	Δ	−0.0057	**−0.019**	−0.011	0.0040	0.0048	−0.0087	−0.0047	−0.021	−0.012
1/6 power law γ	p	0.13	0.13	**0.019**	1	0.69	0.84	0.49	0.16	0.84
	Δ	−0.015	−0.0095	**−0.022**	−0.0040	−0.00061	−0.0016	−0.0058	−0.015	0.0041
Jerk	p	0.69	0.37	0.27	0.16	0.19	0.37	**0.0019**	**0.0019**	**0.0019**
	Δ	−0.071	−0.070	−0.14	0.11	0.21	0.099	**1.22**	**1.4**	**0.86**

**Table 2:** Statistical analysis—Kruskal-Wallis test.

**Metric name**		**Learning**			**Aftereffect**			**Final performance**		
		**NF-PE**	**NF-DF**	**PE-DF**	**NF-PE**	**NF-DF**	**PE-DF**	**NF-PE**	**NF-DF**	**PE-DF**
Exit point error	p model and χ^2^	*p* = 0.80, χ^2^ = 0.43			–			–		
	p	0.84	0.82	1	–	–	–	–	–	–
	Δ	−2.2	−2.3	−0.1	–	–	–	–	–	–
Completion time	p model and χ^2^	*p* = 0.17, χ^2^ = 3.54			*p* = 0.62, χ^2^ = 0.94			*p* = 0.59, χ^2^ = 1.04		
	p	0.93	0.17	0.32	0.64	0.71	0.99	0.69	0.99	0.61
	Δ	−1.4	−7	−5.6	−3.5	−3.1	0.4	−3.2	0.5	3.7
Total Force normalized	p model and χ^2^	*p* = 0.093, χ^2^ = 4.75			–			–		
	p	0.77	0.083	0.31	–	–	–	–	–	–
	Δ	−2.7	−8.4	−5.7	–	–	–	–	–	–
Max force	p model and χ^2^	*p* = 0.44, χ^2^ = 1.63			*p* = 0.26, χ^2^ = 2.65			*p* = 0.11, χ^2^ = 4.26		
	p	0.48	0.53	0.99	0.34	1	0.32	0.32	0.11	0.82
	Δ	4.5	−4.2	0.3	5.5	−0.1	−5.6	−5.6	−7.9	−2.3
Max torque - Z axis	p model and χ^2^	*p* = 0.22, χ^2^ = 3			*p* = 0.91, χ^2^ = 0.17			*p* = 0.92, χ^2^ = 0.15		
	p	0.24	0.35	0.97	0.91	0.99	0.95	0.93	1	0.94
	Δ	−6.3	−5.4	0.9	1.6	0.5	−1.1	−1.4	−0.1	1.3
DTW - force	p model and χ^2^	*p* = 0.34, χ^2^ = 2.11			*p* = 0.27, χ^2^ = 2.60			*p* = 0.14, χ^2^ = 3.86		
	p	0.55	0.34	0.93	0.88	0.51	0.25	0.35	0.13	0.85
	Δ	−4.1	−5.5	−1.4	1.9	−4.3	−6.2	−5.4	−7.5	−2.1
Path length	p model and χ^2^	*p* = 0.052, χ^2^ = 5.91			*p* = 0.41, χ^2^ = 1.77			***p*** **=** **0.028**, **χ^2^** **=** **7.1**		
	p	0.97	0.11	0.069	0.92	0.63	0.39	0.23	0.56	**0.022**
	Δ	0.9	−7.8	−8.7	−1.5	3.6	5.1	−6.4	4	**10.4**
Circle deviation	p model and χ^2^	*p* = 0.87, χ^2^ = 0.266			*p* = 0.20, χ^2^ = 3.19			*p* = 0.11, χ^2^ = 4.41		
	p	0.98	0.86	0.94	0.74	0.55	0.17	0.41	0.69	0.093
	Δ	0.7	2	1.3	−2.9	4.1	7	−5	3.2	8.2
Plane deviation	p model and χ^2^	*p* = 0.44, χ^2^ = 1.61			*p* = 0.21, χ^2^ = 3.07			*p* = 0.10, χ^2^ = 4.45		
	p	0.80	0.41	0.80	0.63	0.67	0.18	0.56	0.51	0.088
	Δ	2.5	5	2.5	−3.6	3.3	6.9	−4	4.3	8.3
Angular path	p model and χ^2^	*p* = 0.26, χ^2^ = 2.66			*p* = 0.32, χ^2^ = 2.25			*p* = 0.11, χ^2^ = 4.33		
	p	0.31	0.99	0.35	0.99	0.42	0.37	0.39	0.72	0.098
	Δ	−5.7	−0.3	5.4	0.4	−4.9	−5.3	5.1	−3	−8.1
DTW - position	p model and χ^2^	*p* = 0.38, χ^2^ = 1.91			*p* = 0.48, χ^2^ = 1.46			*p* = 0.46, χ^2^ = 1.52		
	p	0.85	0.35	0.67	0.63	0.97	0.48	0.55	0.99	0.51
	Δ	−2.1	−5.4	−3.3	3.6	−0.9	−4.5	−4.1	0.2	4.3
1/6 power law α	p model and χ^2^	*p* = 0.88, χ^2^ = 0.23			*p* = 0.68, χ^2^ = 0.75			*p* = 0.67, χ^2^ = 0.77		
	p	0.95	0.88	0.97	0.67	0.81	0.97	0.74	0.99	0.71
	Δ	−1.1	−1.9	−0.8	−3.3	−2.4	0.9	−2.9	0.2	3.1
1/6 power law β	p model and χ^2^	*p* = 0.46, χ^2^ = 1.53			*p* = 0.66, χ^2^ = 0.80			*p* = 0.51, χ^2^ = 1.32		
	p	0.50	0.99	0.56	0.84	0.64	0.94	0.48	0.77	0.89
	Δ	4.4	0.4	−4	2.2	3.5	1.3	4.5	2.7	−1.8
1/6 power law γ	p model and χ^2^	*p* = 0.56, χ^2^ = 1.14			*p* = 0.98, χ^2^ = 0.023			*p* = 0.31, χ^2^ = 2.32		
	p	0.85	0.85	0.53	0.98	0.99	0.99	0.72	0.72	0.28
	Δ	−2.1	2.1	4.2	−0.6	−0.3	0.3	3	−3	−6
Jerk	p model and χ^2^	*p* = 0.88, χ^2^ = 0.235			*p* = 0.37, χ^2^ = 1.94			*p* = 0.060, χ^2^ = 5.61		
	p	1	0.90	0.91	0.55	0.95	0.38	0.81	0.21	0.057
	Δ	0.1	1.7	1.6	−4.1	1.1	5.2	−2.4	6.6	9

**Figure 9:**
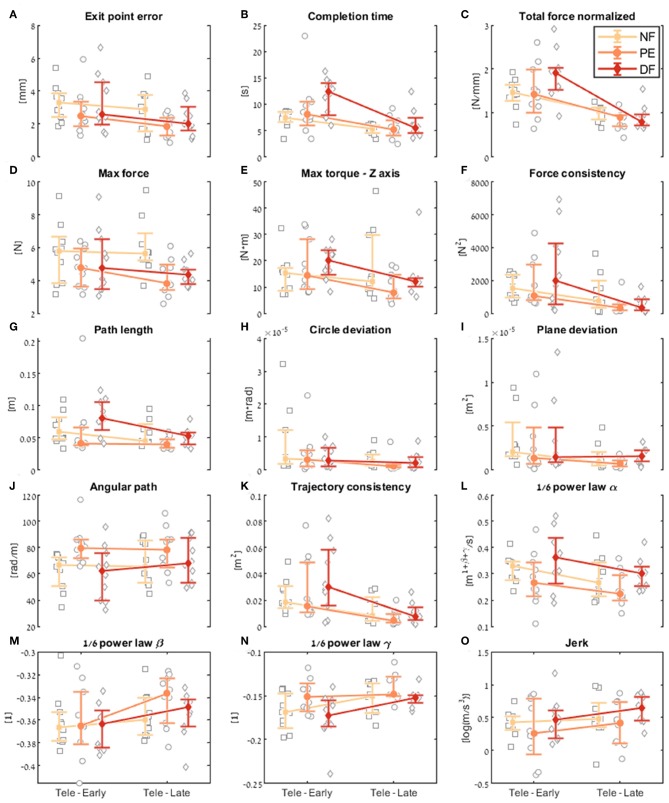
Comparison of the performance of the insertion subtask between early and late dVRK teleoperation. For each metric **(A–O)**, median of first five (early) and last five (late) trials are presented. Each gray marker represents an individual subject. The big marker with error bars shows the median of each group (NF, PE, and DF) and the 95% bootstrap confidence interval, respectively.

**Figure 10:**
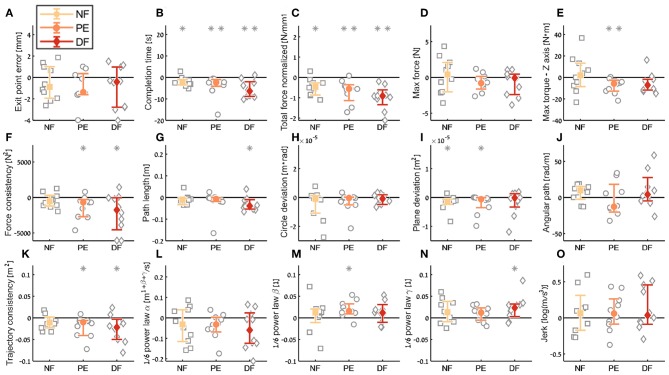
Summary of the dVRK teleoperation *learning* during the insertion subtask. For each metric **(A–O)**, median of difference between late teleoperation stage to early teleoperation stage is presented. Each gray marker represent individual subject. Error bars shows median of each group difference (NF, PE, and DF) and 95% bootstrap confidence interval. Gray asterisks represent the result of a Wilcoxon sign rank test for difference from zero.

First, we examine the *learning during teleoperation* contrast. In Figure 9, we present a detailed look at the results of learning during teleoperation, in addition to the statistical analysis that is summarized in Figure 10. Figure 9 presents the performances of the early stage teleoperation and late stage of teleoperation. For each stage, we present the median and non-parametric bootstrap 95% confidence interval for each of the metrics in each of the teleoperation conditions, along with markers denoting individual participants.

Examining the task performance metrics in teleoperation learning (Figures 9A,B, 10A,B), we found significant improvement in *completion time* (effect of 2–5 s, Table 1) for all conditions, without difference between the feedback conditions (Figure 10B). There was no significant improvement in *exit point error* in teleoperation and no difference between the conditions (Figure 10A). However, the radius of the exit point marker on the tissue was 2 mm, and thus, although we did not see a statistically significant difference between the groups, the force feedback conditions (PE and DF) medians are closer to the marker radius compared to NF condition. Hence, it could be that participants did not try to improve their exit point accuracy more than the marker radius. Another important observation is that the dispersion of exit point error between participants was higher in the tele-early stage compared to tele-late stage (Figure 9A), and this is more prominent in the force feedback conditions (DF and PE). So although there was no improvement in the groups' exit point error median, we think that participants with high exit error at the tele-early stage improved their exit point error accuracy, but we cannot provide quantitative statistical support to this observation.

Looking at the force metrics in teleoperation learning (Figures 9C–F, 10C–F), for the *total normalized force* metric, there was significant learning for all conditions without difference in learning between the three conditions (Figure 10C). All participants reduced their applied forces during teleoperation, and therefore, in real surgery this would reduce the tissue damage. When looking at the *maximum forces*, we did not see significant improvement in none of them (Figure 10D), but when looking at the details (Figure 9D), we can see that at the tele-early stage there was no visible difference between the conditions, and in the tele-late stage the dispersion for the force feedback conditions was reduced and the tendency was toward lowering the maximum forces, while the NF group did not reduce the median of the maximum forces. It is important to note that we did not find significant differences between the two stages in the conditions' median, nor in the interaction factor. In addition, when we looked at the *maximum torque around the vertical axis* (Figure 10E), we saw that only PE feedback condition had significantly reduced its maximum torques, which ideally should be zero. We did not see a significant difference between the conditions, but we did see that the DF feedback condition had at first (Tele-early stage, Figure 9E) higher maximum torques compared to tele-late stage (excluding 1 participant); this might be due to difficulty in stabilizing the system. Although participants did not receive feedback on the torques DOFs, most of them reduced the maximum torques around the vertical axis. As for the consistency of tissue interaction forces, the *force consistency* metric, for both force feedback conditions, DF and PE, we saw a significantly improvement in the consistency of forces in learning during teleoperation (Figure 10F). It is important to note that there was no statistically significant difference in learning between the conditions. Higher consistency of force profiles might indicate a certain technique that participants with force feedback acquired to help them perform the task.

Looking at the kinematics metrics in teleoperation learning (Figures 9G–K, 10G–K), we saw no significant difference between the feedback conditions. The movements of force feedback conditions (PE and DF) became significantly more consistent in the teleoperation learning process (Figure 10K). The *trajectory consistency* for the DF group's median and variability was higher in the tele-early stage compared to the NF and PE conditions, while at the tele-late stage there was no visible difference between the groups (Figure 9K); this might be because participants experienced difficulties in stabilizing the system in the DF condition at the beginning. There was a significant reduction during teleoperation learning in plane deviation for PE and NF (Figure 10I), while there was no learning in DF. All 3 conditions presented lower dispersion in *path length, deviation from circle*, and *deviation from plane* in tele-late stage compared to tele-early stage and there was no visible difference between the performances of the 3 conditions (Figures 10G–I). We saw no learning for the angular path (Figure 10J). Based on the results in Sharon et al. (2017) that showed that participants increased their angular path during the insertion task, we expected to see a similar increase in our study, but this was not the case.

Looking at the motor control oriented metrics in teleoperation learning (Figures 9L–O, 10L–O), participants' movements were generally consistent with the speed-curvature-torsion power law (mean *R*^2^ with standard deviation of the fit: $R_{NF}^{2}=0.80\pm 0.06$, $R_{PE}^{2}=0.79\pm 0.06$, $R_{DF}^{2}=0.81\pm 0.06$), and β and γ parameters had tendency toward their theoretical values (−1/3 and −1/6 respectively). Although there was no significant difference between the two stages, this result indicated that participants tendency was to perform movements that follow the 1/6 power law. In none of the force feedback conditions there was no effect of learning on the velocity gain factor, α, indicating that the participants did not generally increase their movement tempo as they repeated the needle driving task. The jerk of the participants' movements also was not affected by the 3 conditions, nor was it reduced in the late stage. This result indicates that participants movement did not become smoother during teleoperation trials.

**Figure 11:**
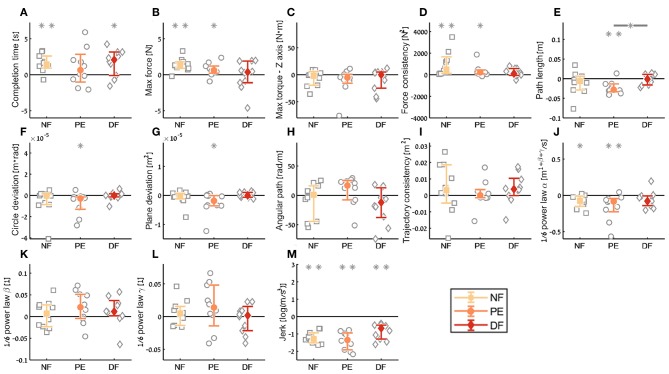
Summary of the dVRK teleoperation *final performance* compared to baseline for the insertion subtask. For each metric **(A–M)**, median of difference between late teleoperation stage to late first open stage is presented. Each gray marker represent individual subject. Error bars shows median of each group difference (NF, PE, and DF) and 95% bootstrap confidence interval. Gray asterisks represent the result of a Wilcoxon sign rank test for difference from zero.

In the *final performance* contrast we quantified the performance at the late stage of teleoperation compared to the baseline performance at the late stage of the first open needle driving. The insertion completion time (Figure 11A) of NF and DF conditions were significantly higher in teleoperation compared to open, while for PE condition there was no significant difference. As for the force metrics, there was no significant difference between the conditions for all force metrics. In the NF and PE conditions, participants applied significant more maximum force (effect size of 1.37 [N] and 0.72, respectively) in tele-late stage compared to open 1-late (Figure 11B), and there was no difference in maximum force for the DF feedback condition. For NF and PE conditions the consistency of forces (Figure 11D) was lower in tele-late stage compared to open-1 late stage, while for the DF condition, there was no difference between the open needle driving and teleoperation needle driving.

In the *final performance* of the kinematic metrics, only for the *path length* (Figure 11E) there was a significant difference between PE and DF conditions: the PE force feedback group performed the task with shorter path length compared to DF force feedback, when looking on the teleoperation needle driving compared to baseline. For the other kinematic metrics, there was no difference between the conditions in the final performance. Similarly, there was no significant difference between the feedback conditions in the motor control oriented metrics. Comparing the final performance in velocity gain factor α metric (Figure 11J), in NF and PE conditions participants movement was slower in teleoperation compared to open needle driving baseline. The jerk (Figure 11M) for all conditions was significantly lower in the teleoperation; this might be because the control system of the dVRK adds damping to participants movements.

**Figure 12:**
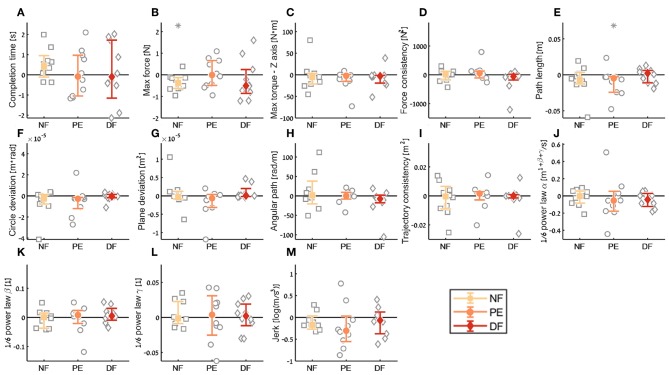
Summary of the open needle driving *aftereffect* for the insertion subtask. For each metric **(A–M)**, median of difference between early stage in the second open needle driving to late stage in the first open needle driving are presented. Each gray marker represent individual subject. Error bars shows median of each group difference (NF, PE, and DF) and 95% bootstrap confidence interval. Gray asterisks represent the result of a Wilcoxon sign rank test for difference from zero.

For the *aftereffect* contrast (Figure 12) we did not see aftereffect in open needle driving for majority of the metrics. This means that the participants performance did not deteriorate due to the 60 trials of the task in teleoperation under any of the feedback conditions, and participants did not improve their performance in open needle driving after training the same task in teleoperation. The fact that the open performance did not deteriorate following teleoperation is encouraging as it suggests that if during surgical cases surgeons have to revert to open cases their performance would continue to be the same as before regardless to the force feedback condition.

## 4. Discussion

We examined the effect of feedback conditions on several aspects of task performance and movement. We used a task of surgical needle driving to evaluate this effect on participants in two setups: (1) teleoperation, as in RAMIS, and (2) using a needle holder, as in open surgery. We designed an experimental setup such that participants performed the task in the two setups while we simultaneously recorded their position, orientation, and tissue interaction forces. In teleoperation, force feedback conditions included two simple force feedback approaches: (1) position exchange force feedback, and (2) direct force feedback sensed from an F/T force sensor. The third condition was no force feedback condition. We designed a protocol to test the *learning during teleoperation*, the *final performance* in teleoperation and the *aftereffect* of teleoperation on open needle driving. The experimental apparatus and protocol we designed enabled us to study different aspects of human movement and force control during complex movement in a surgical task in RAMIS. We developed new metrics to assess the quality of surgical needle driving: trajectory consistency, force consistency, circle deviation, and plane deviation. We also used recently proposed as well as classical metrics to assess performance: exit point error, completion time, total normalized force, max force, max torque *z* axis, path length, angular path, speed-curvature-torsion power law, and jerk.

In a study with 30 participants, we found that in several of the metrics, such as force consistency and maximum applied torques in the vertical direction, the two conditions with force feedback presented statistically significant improvement during teleoperation (i.e., learning). In addition, in some of the metrics, such as task completion time and total normalized force, there was an improvement in all the force feedback conditions. However, when we compared directly between the learning in the three different feedback conditions, none of the effects reached statistical significance. Moreover, we did not find any statistically significant differences between the 3 force feedback conditions in the final performance in teleoperation (except one difference between DF and PE in path length) and the aftereffect. Therefore, we conclude that in our detailed analysis of the performance of needle driving through soft tissue we did not find a benefit to presenting novice participants with force feedback. Hence, our data suggests that the advantage of state of the art force feedback methods to tasks that require interaction with homogeneous soft tissue is questionable.

Up to performing this research, several studies compared conditions with force feedback (usually one algorithm of force feedback) to no force feedback condition, and usually added another condition with the force feedback as visual information (Tholey et al., 2005; Arata et al., 2008; Gwilliam et al., 2009; Santos-Carreras et al., 2010; Talasaz et al., 2017). Results were inconclusive and were task dependent. We expected to find statistically significant differences between the three force feedback conditions, and more specifically, we expected to find worse performance at the end of teleoperation without force feedback compared to the other two force feedback conditions, as in Talasaz et al. (2017), Currie et al. (2017), and Gwilliam et al. (2009), and applied forces in Okamura and Verner (2009) and Santos-Carreras et al. (2010). Surprisingly, we did not see such differences for all the contrasts and metrics except one, consistently with task completion time in Verner and Okamura (2007), Santos-Carreras et al. (2010), and task error in Okamura and Verner (2009).

One possible explanation is that the 3D high-definition visual feedback compensated for the missing feedback in the no force feedback (NF) and the poor haptic feedback in the position exchange force feedback (PE) conditions. The 3D visual compensation is more prominent when interacting with a deformable object such as artificial silicone tissue. Indeed, previous studies showed the importance of 3D vision (Weber and Schneider, 2014). Another explanation might be that after 60 trials in the novel environments in our experiment the human motor system adjusted to the new conditions and perform the same. In Nisky et al. (2015), learning curve of task completion time of non-medical novice participants in needle driving task did not seem to reach a plateau after 80 trials, while for path length it seems that the majority of learning improvement was in the first 20 trials. Another possibility is that 60 trials in teleoperation were not sufficient to the learning of the motor system, and there is potential for further improvement in task performance that would eventually reveal advantages to one of the force feedback conditions. This explanation is consistent with Nisky et al. (2014b), where learning was observed even after 200 trials in a simple reaching task in teleoperation.

Another possible explanation for our failure to find statistically significant differences between the force feedback conditions might be the high between participants variability. This variability could be a consequence of the location of needle grasping, or variability in entrance and exit points. Large variability in the sample reduces the power of statistical tests. This indicates that larger sample sizes or adding constraints on the task may be useful to increase power in future studies. To partially mitigate the influence of between-participants variability, for each contrast we compared the performance of the participants to their relevant baseline performance. One more reason for the large variability is the human movement variability, which allows to successfully perform a given task with different trajectories. Generally, variability is unavoidable in the motor system, and is even considered a virtue of the sensorimotor system (Touwen, 1993; Todorov and Jordan, 2002; Faisal et al., 2008; Stergiou and Decker, 2011; Kaipust et al., 2012; Nisky et al., 2014a; Buzzi et al., 2019). With repetitions of the task, the decrease in trajectory consistency and force consistency metrics showed that the within-participant variability in consecutive trials was lower at the late stage of the experiment. This suggests that unlike in simpler movements (Müller and Sternad, 2004; Dingwell et al., 2012), participants gradually progressed toward adopting a stereotypic trajectory to perform the task.

In our experiment, we implemented two force feedback conditions, (1) position exchange force feedback and (2) direct sensing from a force sensor that was attached to the tissue plate. In addition, all participants in all 3 conditions received gravity compensation as described in (Chen et al., 2017), and more advanced algorithms (e.g., the one implemented by Lin et al., 2019) could improve the performance. It is possible that with better gravity compensation the participants could perform better and this could have allowed for observing more pronounced differences between the different force feedback conditions.

In position exchange force feedback, we estimated the applied forces based on the error between desired and current state of the robot tooltip (Siciliano and Khatib, 2016). While we did not perform an analytic analysis of stability in our force feedback implementations, we could easily see that the position exchange force feedback remained stable throughout the experiment. However, the force feedback did not feel real, as the forces when interacting with the tissue felt lower compared to DF condition and open needle driving. The position exchange form of force feedback is better when interacting with a stiff object rather than a soft object. Since the interaction in our experiment is with a deformable tissue, the forces felt less realistic and smaller than the actual applied forces. In addition, because there was no dynamic compensation, participants felt forces when moving the tool freely without any interaction with an object. This two issues likely resulted in a poor quality of force feedback in the PE condition.

In the direct force feedback condition, participants received the forces directly from a force sensor located under the tissue. The force sensor recorded the tissue interaction forces with high accuracy, and as a result, the forces felt more realistic than in the PE condition. However, occasionally, we observed the tool starting to get out of stability. To mitigate the instability problem, we empirically tuned a gain of 0.7 for the direct force feedback. In most cases, participants succeed to stabilize the system, and if they did not succeed they stopped teleoperation for a brief moment and returned to the task; however, in some cases, participants were still not able to stabilize the system. In some metrics, the DF condition median was visibly different compared to the PE and NF condition at the early stage trials in teleoperation (e.g., completion time, force consistency, and trajectory consistency), but the difference was not visible in the late stage.

In the *final performance* contrast, our aim was to compare the performances between the three conditions at the late stage in teleoperation. We chose to normalize each participants performances to his or her baseline performances at the late stage of open needle driving. As a result, in this contrast, we compared between the performances in open needle driving to the performances in teleoperation. Comparison between the two setups, in which the participants perform the task with different tools, is not trivial. However, because we compare the way the human operator manipulates the tool end effector (i.e., needle holder tooltip and dVRK large needle driver tooltip), and the metrics we used are relevant for the end effector we believe that this kind of comparison is valid. We did not see statistically significant difference between the feedback conditions in *final performance* (except one difference in path length metric), but we did see that for all metrics the jerk in the teleoperation was lower compared to open needle driving and that forces were for most cases similar in teleoperation late stage and open needle driving.

## 5. Conclusion

In the current study, we failed to find statistically significant differences between the feedback conditions in needle driving through soft tissue. Both our implementations of simple force feedback algorithms did not significantly improve the kinematics and tissue interaction forces of the human operator compared to no force feedback, and there was no difference between the two force feedback algorithms. This may be a result of the poor quality of our force feedback algorithms for soft tissue interaction, or due to sufficient force cues available from visual information about tissue deformation.

Detailed understanding of the effect of haptic feedback on the operators movement and tissue interaction forces is important for developing future guidelines for human-centered design of teleoperated RAMIS systems with force feedback that will eventually provide advantage over unilateral teleoperation. We think that better, human-centered, force feedback algorithms have the potential to improve performance and movement quality compared to no feedback at all or algorithms that don't consider the human operator as part of the control loop.

## 6. Future Work

In the future, it will be interesting to perform a similar experiment with experienced and novice surgeons that are familiar with surgery and RAMIS and to see if one of the force feedback condition will be beneficial for them. In this study, we implemented two simple force feedback algorithms and in future studies, it is important to investigate the performance of participants with other force feedback algorithms (Mahvash and Okamura, 2007; Santos-Carreras et al., 2010; Anooshahpour et al., 2014; Koehn and Kuchenbecker, 2015; Rivero et al., 2017; Quek et al., 2018). Another aspect future studies is to investigate if the high-resolution visual feedback was indeed responsible for the lack of differences between the conditions. It is possible to perform similar experiment with different vision conditions, and particularly the 3D vision compared to 2D vision; To investigate the learning curves and perform the research with more trials; to add more constraints to the needle driving complex task; or to give feedback on performances in real time that will encourage the participants to perform better in the task.

## Data Availability Statement

The datasets generated for this study are available on request to the corresponding author.

## Ethics Statement

The studies involving human participants were reviewed and approved by Human Subject Research Committee of Ben Gurion University of the Negev, Beer-Sheva, Israel. The patients/participants provided their written informed consent to participate in this study.

## Author Contributions

LB designed and built the experimental setup, designed and preformed the experiments, analyzed the data, interpreted the results, and wrote the manuscript. LB and YS assembled and integrated the hardware and software of the dVRK. IN conceived the research idea, designed the experimental setup and experiments, interpreted the results, supervised the work, and reviewed and edited the manuscript. All authors contributed to writing the manuscript, manuscript revision, read, and approved the submitted version.

### Conflict of Interest

The authors declare that the research was conducted in the absence of any commercial or financial relationships that could be construed as a potential conflict of interest.
